# Molecular profiling of colorectal pulmonary metastases and primary tumours: implications for targeted treatment

**DOI:** 10.18632/oncotarget.17048

**Published:** 2017-04-11

**Authors:** Sing Y. Moorcraft, Thomas Jones, Brian A. Walker, George Ladas, Eleftheria Kalaitzaki, Lina Yuan, Ruwaida Begum, Zakaria Eltahir, Andrew Wotherspoon, Angeles Montero-Fernandez, Larissa S. Teixeira Mendes, David Gonzalez de Castro, Sanna Hulkki Wilson, Paula Proszek, Ye M. To, Eliza Hawkes, Amitesh Roy, David Cunningham, Sheela Rao, David Watkins, Naureen Starling, Anne M. Bowcock, Ian Chau

**Affiliations:** ^1^ The Royal Marsden NHS Foundation Trust, London and Sutton, United Kingdom; ^2^ The Royal Brompton and Harefield NHS Foundation Trust, London, United Kingdom; ^3^ National Heart and Lung Institute, Imperial College, London, United Kingdom

**Keywords:** colorectal cancer, heterogeneity, metastasectomy, pulmonary metastases, RAS

## Abstract

This study aimed to molecularly characterise colorectal pulmonary metastases (PM) and investigate whether their molecular profiles were concordant with those of the primary tumour. Clinical data and archival formalin fixed paraffin embedded tissue samples were retrospectively collected from patients who underwent ≥ 1 pulmonary metastasectomies for colorectal cancer between 1997–2012. Primary tumour and metastatic samples were analysed using a targeted capture sequencing panel of 46 cancer-associated genes. The 5-year progression-free and overall survival rates for the 81 patients in this study were 32% (95% CI 22–42%) and 77% (95% CI 66–85%) respectively. Fifty-four patients had samples available from ≥ 1 PM, and sequencing data were successfully obtained from 33 PM from 24 patients. The most frequently mutated genes were *APC* (71%), *KRAS* (58%) and *TP53* (46%). Seventy-three percent of the 15 patients with matched primary and PM samples and 6 of the 7 patients (86%) with data from ≥ 2 PM had concordant molecular profiles. The concordance for *KRAS* and *NRAS* was 100%. At our institutions, patients with resectable colorectal PM had a favourable prognosis. RAS mutations were commonly detected in PM and the molecular profiles of colorectal PM were highly concordant with the primary tumour.

## INTRODUCTION

Pulmonary metastases (PM) occur in approximately 10–20% of patients with colorectal cancer [1–4]. The majority of patients with PM are treated with palliative intent and have a poor prognosis, but pulmonary metastasectomy may be a curative option for carefully selected patients with limited sites of disease. However, up to 68% of patients develop recurrent disease following initial metastasectomy [5, 6] and although selected patients with operable metastases may benefit from multiple re-resections [7, 8], many patients will ultimately develop incurable disease.

Precision medicine involves selecting treatment based on the molecular characteristics of an individual patient’s tumour. In metastatic/advanced colorectal cancer, a patient’s suitability for treatment with anti-EGFR monoclonal antibodies is determined by the presence of mutations in *KRAS* and/or *NRAS* [9]. However, discordance between the molecular profiles of the primary tumour and metastases can occur due to heterogeneity of the primary tumour, the progression of specific tumour subclones, the gain or loss of mutations during disease progression or technical issues (such as low DNA quality) leading to inaccurate results [10, 11]. Tumour heterogeneity may limit the efficacy of targeted therapies and therefore it is essential to establish whether there are significant molecular differences between primary tumours and metastatic sites of disease.

*KRAS* mutation status has been shown to be highly concordant between the primary colorectal tumour and hepatic metastases [10, 12]. It has therefore been suggested that *RAS* results from analysis of the primary tumour are sufficient for clinical decision making and that additional *RAS* testing of metastatic sites is unnecessary. However, there is a paucity of data available on PM as the majority of previous studies focus on hepatic metastases. It is currently unclear if results from studies on hepatic metastases can be applied to metastases occurring at other sites, and there is some evidence that extra-hepatic metastases are more likely to show discordant molecular results [10]. To investigate this we performed next generation sequencing (NGS) on colorectal pulmonary metastasis samples to look for alterations in 46 cancer-associated genes and compared the molecular profiles of the PM with those of the primary tumour.

## RESULTS

### Patient characteristics and clinical outcomes

Eighty-one patients underwent 121 pulmonary metastasectomies. The baseline characteristics of these patients are shown in Table 1. Nineteen patients (23%) presented with synchronous metastases at the time of diagnosis. The details of the metastasectomies are shown in Supplementary Table 1. Eighty-eight of the 121 metastasectomies (73%) were for unilateral metastases, neoadjuvant chemotherapy was given prior to 58 (48%) of metastasectomies and all targeted therapies were given in combination with systemic chemotherapy. Mediastinal dissection was not routinely performed.

**Table 1:** Patient characteristics (*n* = 81)

**Characteristic**	***N* (%)**
Gender	
Male	50 (62)
Female	31 (38)
Median age (interquartile range) at diagnosis, years	62 (54–67)
Site of primary tumour	
Caecum/ascending colon	7 (9)
Transverse colon	3 (4)
Sigmoid/rectosigmoid	25 (31)
Rectum	42 (52)
Colorectal (site not specified)	4 (5)
Stage at diagnosis	
I	3 (4)
II	19 (24)
III	38 (47)
IV	19 (24)
unknown	2 (3)
Differentiation	
Well	1 (1)
Moderate	64 (79)
Poor	5 (6)
Unknown	11 (14)
Extramural venous invasion (EMVI)	
No	27 (33)
Yes	18 (22)
Unknown	36 (44)
Neoadjuvant treatment of primary tumour	
Neoadjuvant chemoradiotherapy	13 (16)
Neoadjuvant chemotherapy	6 (7)
Neoadjuvant chemotherapy and chemoradiotherapy	12 (15)
Neoadjuvant radiotherapy	2 (3)
Adjuvant treatment of primary tumour	
Adjuvant chemotherapy	57 (70)
Adjuvant chemoradiotherapy	3 (4)
Site of first metastasis	
Lung only	60 (74)
Liver only	14 (17)
Lung and other site^1^	5 (6)
Median time from diagnosis to first metastasis at any site (IQR), months	16 (1–28)
Median time from diagnosis to first pulmonary metastasis (IQR), months	19 (2–29)
Number of pulmonary metastasectomies per patient^2^	
1	52 (64)
2	22 (27)
3	4 (5)
4	2 (3)
5	1 (1)

^1^Other sites: lymph node, peritoneum, liver.

^2^Staged metastasectomies were performed in 23 patients with bilateral metastases and were counted as one metastasectomy as no other treatment was administered between each surgical procedure.

The median follow-up was 87 months (interquartile range (IQR) 72–113 months) and at the time of last follow-up, 23 patients (28%) remained disease-free. The in-hospital and 30-day mortality after metastastectomy was 0%. The median progression-free survival (PFS) and overall survival (OS) were 24 months (95% CI 19–38) and 91 months (95% CI 72–131) respectively, and the 5-year PFS and OS rates were 32% (95% CI 22–42%) and 77% (95% CI 66–85%). Twelve patients (15%) also had PM treated by radiofrequency ablation (RFA) (1-4 separate RFA procedures per patient), 38 patients (47%) received ≥ 1 lines of palliative chemotherapy and 28 patients (35%) had ≥ 1 resections of non-PM (mainly liver metastases).

The number of resections (2+ vs. 1, HR 4.12 95% CI 2.35–7.23; *p* < 0.001) and the size of the largest pulmonary metastasis (> 11 mm vs. ≤ 11 mm, HR 0.53, *p* = 0.02, 95% CI 0.31–0.92; *p* = 0.02) were the only two factors associated with PFS. No factors were associated with OS.

### Molecular characteristics of pulmonary metastases

**Figure 1:**
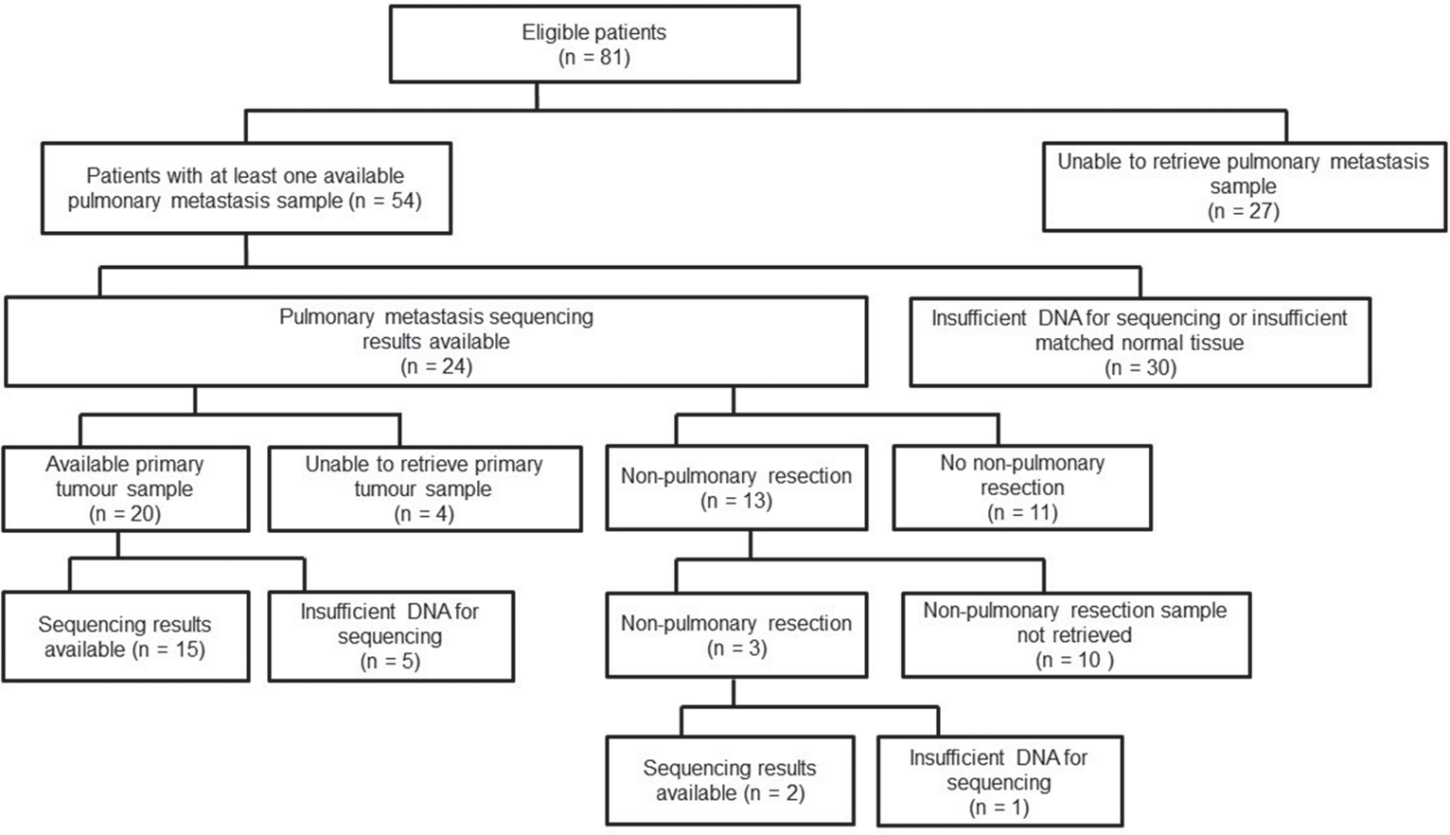
Availability of primary tumour and metastasectomy samples

Figure 1 shows the availability of primary tumour and metastasectomy samples and whether these samples were successfully sequenced. One or more pulmonary metastasectomy samples were available for 54 patients (67%). Sequencing data were available for 33 PM from 24 patients (6 patients had results for 2 PM and 1 patient for 4 PM). Of these 24 patients, sequencing data were also available for the primary tumour for 15 patients and from another metastatic site in 2 patients.

The most frequently altered genes in the first available PM sample for each patient were *APC* (*n =* 17, 71%), *KRAS* (*n =* 14, 58%) and *TP53* (*n =* 11, 46%) and further details of these alterations are shown in Supplementary Figures 1–3 and Supplementary Table 2 (in some samples > 1 variant for each gene was detected). Mutations were also detected in *PIK3CA* (*n =* 5, 21%), *FBXW7* (*n =* 4, 17%), *CTNNB1* (*n =* 3, 13%) and *TCF7L2* (*n =* 3, 13%). Mutations in *ATM, NRAS* and *PTEN* were detected in 2 samples each and mutations in *ARID1A*, *BRAF, CDKN2B*, *ERBB4*, *NOTCH3* and *RET* in 1 sample each. *RAS* mutations were detected in 15 of the 24 patients (63%) with sequencing results from PM samples (*KRAS* mutant/*NRAS* wild-type: 13 patients, *NRAS* mutant/*KRAS* wild-type: 1 patient, *KRAS* mutant/*NRAS* mutant: 1 patient). No amplifications or translocations were detected in the genes that were interrogated by our assay.

### Concordance between the molecular characteristics of the primary tumour and matched pulmonary metastases

Eleven (73%) of the 15 patients with matched primary and PM samples had concordant molecular profiles and of the 7 patients with more than one PM sample, 6 (86%) had concordant molecular profiles (see Table 2). An example of the results for an individual patient is shown in Supplementary Figure 4. The molecular profiles of the two non-pulmonary resection samples (one liver and one pancreatic metastasis) were concordant with the respective primary tumours.

**Table 2:** Concordance between the molecular characteristics of the primary tumour and matched lung metastases by NGS

Patient no.	Stage at diagnosis	Genes containing alterations (mutations, insertions or deletions)						All samples concordant
		**Primary tumour**	**1st pulmonary metastasis**	**2nd pulmonary metastasis**	**3rd pulmonary metastasis**	**4th pulmonary metastasis**	**Other metastasis**	
002	T4 N0 M0	APC, KRAS, PIK3CA	APC, KRAS, PIK3CA	No variants detected	-	-	-	No^1^
005	T3 N0 M0	FBXW7	FBXW7	-	-	-	FBXW7	Yes
007	T2 N1 M0	TP53, ARID1A	TP53, ARID1A	-	-	-	-	Yes
011	T3 N2 M0	APC, KRAS, TP53, FBXW7	APC, KRAS, TP53, FBXW7	-	-	-	-	Yes
019	T3 N0 M0	NRAS, PIK3CA, PTEN, SMAD4	NRAS, PIK3CA, PTEN, SMAD4	-	-	-	-	Yes
021	T2 N0 M0	No sample available	APC, KRAS	APC, KRAS	-	-	-	Yes
025	T3 N1 M0	APC, KRAS	APC, KRAS	-	-	-	-	Yes
032	T3 N1 M0	APC, KRAS, TP53, CTNNB1, NOTCH3	APC, KRAS, TP53, CTNNB1, NOTCH3	-	-	-	-	Yes
033^2^	T2 N0 M0	No sample available	APC, KRAS, FBXW7	APC, KRAS, FBXW7	APC, KRAS, FBXW7	APC, KRAS, FBXW7	-	Yes
037	T1 N1 M0	APC (2 mutations), KRAS, PIK3CA, TP53	APC (1 APC deletion not detected), KRAS, PIK3CA, TP53	-	-	-	-	No^1^
038^2^	T3 N2 M0	No sample available	APC, BRAF, TP53, CDKN2B	APC, BRAF, TP53,CDKN2B	-	-	-	Yes
040	T3 N1 M0	APC, KRAS	APC, KRAS	-	-	-	APC, KRAS	Yes
041	T3 N1 M0	APC, TP53, ATM, CTNNB1	APC, TP53, ATM, CTNNB1	APC, TP53, ATM, CTNNB1	-	-	-	Yes
045	T4 N0 M1	APC	No variants detected	-	-	-	-	No^1^
050	T3 N2 M0	APC, KRAS, TP53, TCF7L2	APC, KRAS, TP53, TCF7L2	-	-	-	-	Yes
051	T3 N0 M0	APC, KRAS, PIK3CA, TP53, AKT1, SMAD4, TCF7L2	APC, KRAS, TP53, TCF7L2 (AKT1, PIK3CA, SMAD4 mutations not detected)	-	-	-	-	No^1^
052	T2 N0 M1	KRAS, NRAS, PIK3CA, TP53, CTNNB1	KRAS, NRAS, PIK3CA, TP53, CTNNB1	-	-	-	-	Yes
056	T3 N1 M0	No variants detected	No variants detected	No variants detected	-	-	-	Yes
063^2^	T3 N2 M0	No sample available	APC, TP53	APC, TP53	-	-	-	Yes

^1^ddPCR for PIK3CA and KRAS on the 2nd pulmonary metastasis sample for patient 002 detected low frequency mutations in these genes. ddPCR was not performed for APC in patient 002 or the other discordant samples as probes were not available for the other variants. Discordant results are likely to be due to low tumour content of these samples. ^2^Patients 033, 038, and 063 had bilateral pulmonary metastases. 033: 1st pulmonary metastasis = left lung, 2nd pulmonary metastasis = right lung, 3rd pulmonary metastasis = left lung (2nd resection), 4th pulmonary metastasis = right lung (2nd resection). 038 and 063: 1st pulmonary metastasis = left lung, 2nd pulmonary metastasis = right lung.

**Figure 2:**
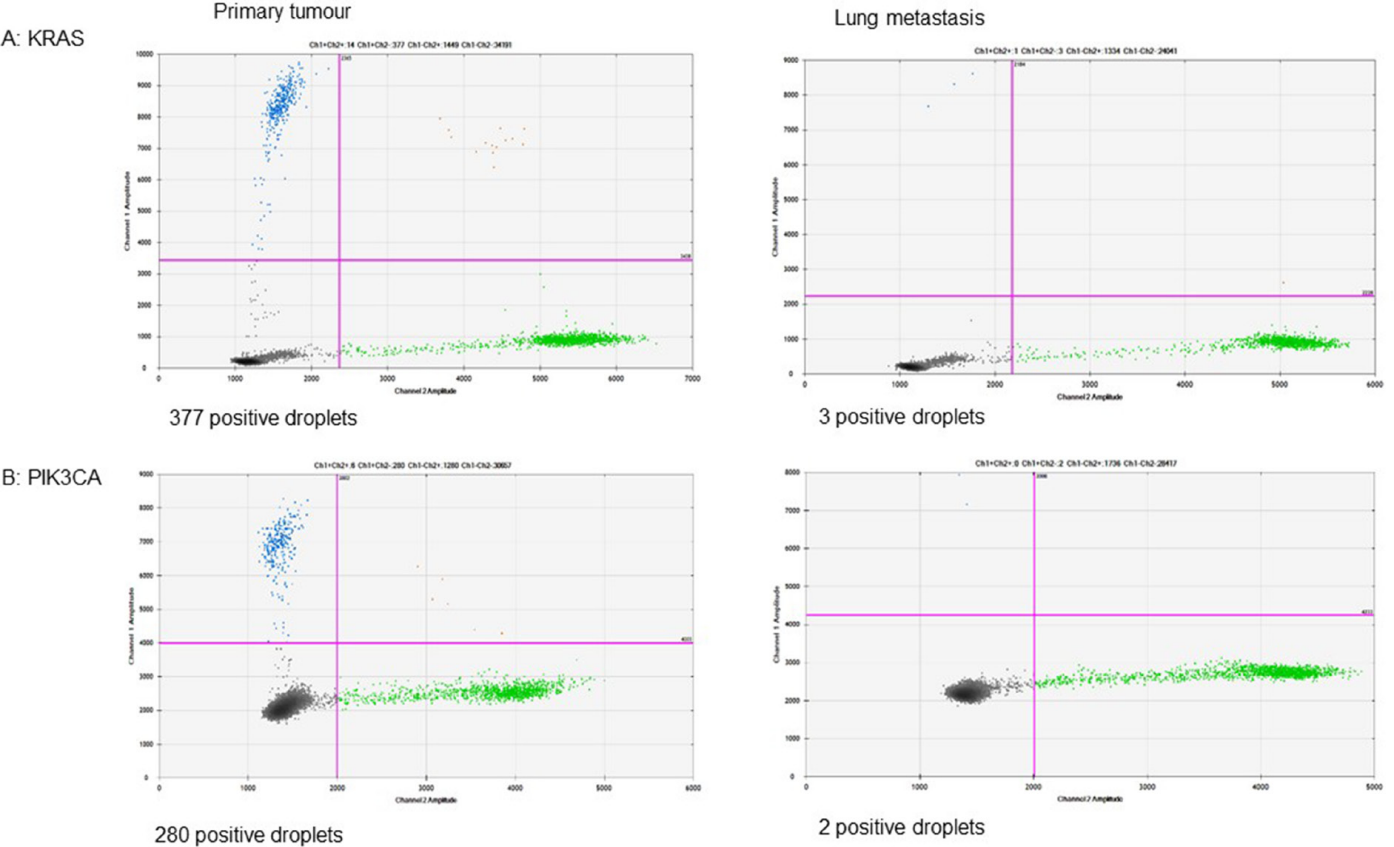
Digital PCR detected mutations that were below the level of detection by NGS in patient 002

In patients with sequencing data from matched samples, the concordance between the primary tumour and the PM for *KRAS*, *NRAS* and *BRAF* was 93% (14/15), 100% (15/15) and 100% (14/14 patients) respectively. *BRAF* results were not successfully obtained from a matched PM sample in 1 patient due to suboptimal coverage. The patient with discordant *KRAS* results (patient 002) had a *KRAS* mutation detected in their primary tumour and first PM, but this mutation was not detected in their second PM using NGS. The most likely reason for this is that the second PM was highly necrotic and had a very low tumour content. Digital droplet polymerase chain reaction (ddPCR) of the primary tumour and second PM samples for this patient (see Figure 2) showed that *KRAS* and *PIK3CA* mutations occurred in the primary tumour at a higher fractional abundance than in the second PM (20.8 versus 0.29 for *KRAS* and 18.0 versus 0.11 for *PIK3CA* respectively). This patient did not receive any systemic anti-cancer therapy between the first and second pulmonary metastasectomies. Three other patients had discordant results. Two of these patients (patients 037 and 045) had an *APC* variant detected in the primary tumour but not in the PM sample whilst for the third patient (patient 051) *PIK3CA*, *AKT1* and *SMAD4* mutations were detected in the primary tumour but not in the PM sample. These discordant results are likely to have been due to the low tumour content of the corresponding pulmonary metastasectomy samples (< 10–20%), so any mutations present may have fallen below the level of detection using NGS. Between the date the primary tumour was resected and the date of pulmonary metastasectomy, patients 037 and 045 received chemotherapy with capecitabine and oxaliplatin and patient 051 received chemotherapy with capecitabine (adjuvant treatment) and then 5-fluorouracil and irinotecan (neoadjuvant treatment).

## DISCUSSION

This study provides information on both the clinical and molecular characteristics of patients with colorectal PM. In our series, there was a high proportion of patients with rectal tumours and 63% of patients were *RAS* mutant. This is in keeping with previously published data that suggested an association between both rectal tumours and *KRAS* mutations and the development of PM [1, 13–15], although the *KRAS* mutation rates previously reported range from 36–62% for PM samples [13, 16–18]. Other studies have also shown that the presence of *KRAS* mutations is a poor prognostic factor both in colorectal cancer in general and specifically in patients with colorectal lung metastases [16, 18, 19], although this may be partly due to patients with *RAS* mutations being unsuitable for treatment with anti-EGFR monoclonal antibodies.

Our patients had a 5-year OS rate of 77%, which is higher than the 5-year OS rates of 40–68% reported by other series [7, 20]. Due to the retrospective nature of our study and the limited availability of samples there are multiple potential confounding factors that may account for the differences in patient outcomes, including careful patient selection, however it is noteworthy that many of our patients were aggressively treated, with many patients undergoing multiple resections (both for PM and for other sites of disease).

Our study is one of only a few studies that have characterised the molecular profiles of colorectal PM and in general, the molecular profiles were consistent with those expected for primary colorectal cancer [21]. Currently, the most clinically significant mutated genes in colorectal cancer are *KRAS*, *NRAS* and *BRAF* as these genes provide information on a patients’ prognosis and their suitability for anti-EGFR therapies [9, 22]. Previous data have suggested that the status of these genes is highly concordant between the primary tumour and metastases, with concordance rates of 92% for KRAS and 97% for BRAF [23]. The majority of these data are based on studies of hepatic metastases and extra-hepatic sites appear to have a lower concordance rate (95% concordance for hepatic metastases vs 86% for extra-hepatic metastases, *p* = 0.01) [10]. Similarly, Kim et al performed automated sequencing using capillary electrophoresis technology (ABI 3130xl Genetic Analyzer) and reported that PM have a high discordance rate for *KRAS* status (32.4%, 12/37, *p* = 0.014) [14]. In our patients, the concordance (by NGS and ddPCR) between the primary tumour and PM was 100% for *KRAS*, *NRAS* and *BRAF*. PM samples have a low tumour content and so our higher than expected concordance rates may be at least partly due to advances in sequencing methodology compared to previous studies.

The discordant results we obtained in the primary versus PMs are likely to have been due to the technical constraints of our NGS panel and the low tumour content of the PM samples. For example, variants may have been present at a frequency of < 5% and were therefore below the ≥ 5% variant allele cut-off used to call mutations using the NGS panel. This hypothesis is supported by the results of the ddPCR experiments in patient 002. In this patient, the PM sample was highly necrotic and had a low tumour content. Our NGS panel did not therefore have the sensitivity to detect mutations in the PM sample, but ddPCR (which has a higher sensitivity) detected mutations in *KRAS* and *PIK3CA* that were below the level of detection by NGS.

The majority of previous studies investigating the concordance of the mutational profile of metastatic sites with that of the primary tumour focused on a limited number of genes (e.g. *KRAS*). Studies that performed a more comprehensive molecular analysis have revealed a more complex picture. For example, Danner *et al.* performed comparative genomic hybridisation on colorectal tumours and matched PM and demonstrated that PM were more likely to have losses in 5q (26% vs 3%, *p* = 0.012) [24] where *APC* resides. Similarly, Vermaat *et al.* analysed 1,624 cancer-associated genes in matched primary tumours and hepatic metastases from 21 patients using targeted deep-sequencing [25]. This study showed that the hepatic metastases gained an average of 83 potentially function-impairing variations and lost an average of 70 variations, including abnormalities in genes encoding components of pathways such as the EGFR/PI3K/VEGF pathway [25]. Our study is one of the most detailed analyses of colorectal PM performed to date (investigating mutations and copy number variations), and we have shown that PM have similar molecular profiles to those of the primary tumour. It is possible that this is because we analysed 46 genes that are known to develop a mutation early in tumorigenesis. A more unbiased analysis may detect greater differences between the primary tumour and the PM, but these may not necessarily be therapeutically meaningful.

The results of our study are limited by the small number of patients for whom molecular results were available from both the primary tumour and a PM. It was not possible to locate a high proportion of samples due to the long inclusion period of 15 years and as no blood samples were available, the only source of germline DNA was normal tissue samples and these were frequently not available. In addition, due to the retrospective nature of this study, all DNA samples were formalin fixed paraffin embedded (FFPE) derived and some of the original primary tumour resections were almost 20 years old (although the date of resection did not appear to influence the sequencing success rate). The gene panel required a substantial amount of DNA (200–400 ng), and many samples did not yield sufficient DNA for analysis. There were a number of reasons for this, including exhaustion of samples by previous standard-of-care analyses or use in other clinical trials, a previous unsuccessful attempt to perform exome sequencing on these samples (which failed as DNA extracted using Cobas resulted in poor quality libraries and led to all the samples being re-extracted using Qiagen) and tumour regression in resection samples due to neoadjuvant chemotherapy/chemoradiotherapy. However, this study also provides important information on the feasibility of future genomic studies in similar patient cohorts.

In summary, patients treated for resectable colorectal PM at our institutions had a favourable prognosis. After adjusting for tumour content, the molecular profiles of the PM were highly concordant with those of the primary tumour with respect to the 46 genes included in the NGS panel and so *RAS* results from the primary tumour may be sufficient for clinical decision-making regarding the use of anti-EGFR therapy.

## MATERIALS AND METHODS

### Patients

After approval from a research ethics committee (REC reference 12/SC/0158), surgical records were used to identify patients who underwent one or more pulmonary metastasectomies for colorectal adenocarcinoma with curative intent between 1997 and 2012 at the Royal Brompton and Harefield NHS Foundation Trust. Only patients who had received treatment at the Royal Marsden and patients whose pulmonary nodules were found to be related to their colorectal adenocarcinoma were included. Clinical information, including patient demographics, clinical and pathological characteristics, treatment details and patient outcomes were retrospectively collected from electronic patient records.

### Sequencing methods

Archival FFPE tissue samples surplus to clinical requirements were collected from patients’ colorectal primary tumours, pulmonary metastasectomy specimens and any other resected metastases (e.g. liver metastases). No blood samples were available hence representative areas of normal tissue (e.g. from resected colon or pulmonary specimens) were selected to provide germline DNA for comparison.

5 × 10 μm unstained slides and two haematoxylin and eosin stained slides were cut from FFPE blocks. Tumour content and purity were assessed using the stained slides and the tumour areas were marked by experienced pathologists. Unstained slides were macrodissected, if necessary, and DNA extracted using the QIAamp DNA FFPE Tissue kit (Qiagen). DNA was assessed for quality using the Genomic DNA Analysis ScreenTape on the 2200 TapeStation (Agilent). Control sample DNA was extracted from FFPE tissue using the QIAamp DNA FFPE Tissue kit (Qiagen). DNA samples were quantified using Qubit High Sensitivity kit (Life Technologies). 200 ng of DNA with an average molecular weight > 1000 bp or 400 ng of DNA with an average molecular weight < 1000 bp (both tumour and control) were used in the HyperPlus kit (KAPA Biosystems) to generate sequencing libraries. Libraries from 2–3 patients were combined with a positive control (Human Male genomic DNA (Promega)) and non-template control and hybridisation capture was performed using SeqCap EZ Choice Enrichment kit (Nimblegen). The enrichment kit was designed against 46 genes and details of the gene panel are shown in Table 3. The genes in the panel were chosen because they were of prognostic or predictive significance, were targets in current/forthcoming clinical trials in gastrointestinal cancers or were known to be recurrently mutated in gastrointestinal cancers. As all the samples were FFPE they were all combined in a single pool alongside a positive (Promega) and non-template control. Samples were pooled according to trial identifier (typically 2–3 sample sets per pool) resulting in pools between 3–7 samples and controls (most pools contained 7 samples). The single pool containing all the samples was then hybridised. Combined libraries were sequenced on a MiSeq instrument (Illumina) with 2 × 76 bp reads using MiSeq reagents v3 chemistry.

**Table 3:** Genes included in the targeted capture panel

Mutations						
AKT	APC	ARID1A	ATM	BRAF	CDK4	CDKN2A/B
CTNNB1	DOCK2	EGFR	ELMO1	ERBB2/4	FBXW7	HRAS
IDH1/2	JAK3	KIT	KRAS	MAP2K1/2	NOTCH1/2/3	NRAS
PDGFRA	PIK3CA	PTEN	RET	ROS1	SMAD4	TCF7L2
TP53	UGT1A1	VHL				
**Copy number amplification**						
ALK	CCND1	CDK4/6	EGFR	ERBB2	FGFR2	IGF1/2
KRAS	MET	MST1R	PIK3CA	TRIM44		
**Translocations**						
ALK	FGFR2	RET	ROS1			

The gene panel and its analytical procedures had been validated to the level required for routine clinical use in a Clinical Pathology Accredited laboratory. The validation process involved analysing 50 colorectal tumour tissue samples collected as part of a previous study (the Screening Study of Genetic Changes in Colorectal Cancer), which had established a database of tissue and blood samples from patients with colorectal cancer. These samples had previously been analysed for *KRAS, BRAF, NRAS, PIK3CA* and *TP53* mutations using either Capillary Electrophoresis-Single Strand Conformational Analysis (CE-SSCA), a COBAS polymerase chain reaction (PCR) based mutation test or Tru-Seq Custom Amplicon next generation sequencing (TSCA NGS). Twenty-seven patients were known to have *KRAS* mutations, 13 had *BRAF* mutations, 12 had *PIK3CA* mutations and 2 had *NRAS* mutations. The results obtained from the gene panel were compared with the known mutational profile of these samples and the sensitivity and specificity was 100% for all genes with the exception of *PIK3CA*, which had a specificity of 89.5%. This lower specificity was due to the detection of 8 additional mutations that had not previously been detected and 4 mutations which were below the threshold frequency for detection by previous methods (mutation frequency ranged from 1.94–9.55%). Many of these mutations had not been previously reported or were very rare. The other genes in the panel were not validated as there were no gold-standard test results for these genes, and therefore validation of these genes was extrapolated from the validation of *KRAS, BRAF, NRAS* and *PIK3CA*. The gene panel had also been used to analyse samples from 136 patients with gastrointestinal malignancies who had been recruited into a molecular profiling feasibility study (ClinicalTrials.gov identifier NCT02112357).

### Analysis

The primary analysis was performed through MiSeq Reporter (v2.5.1; Illumina) to generate binary alignment map (bam), coverage and variant files. The secondary analysis was performed through an in-house Molecular Diagnostics Information Management System to generate QC, variant annotation, data visualisation and a clinical report. In the Molecular Diagnostics Information Management System, the bam files were taken and de-duplicated using Picard to generate metrics for each region in the panel of interest. Oncotator (v1.5.3.0) was used to annotate point mutations and indels. Annotations include gene names, functional consequence (e.g. Missense), PolyPhen-2 predictions, and cancer-specific annotations from resources such as COSMIC, Tumorscape, and published MutSig results. All potential mutations and copy number variations were visualised using Integrative Genomics Viewer and two scientists were required to analyse the results and review the mutation report blindly and independently.

### Digital droplet polymerase chain reaction

ddPCR of FFPE tumour derived DNA from a patient with discordant molecular results was performed using the QX200 ddPCR system (BioRad). TaqMan assays were designed to match mutations of interest and validated on a positive sample prior to use. The assays were performed using ddPCR Supermix for Probes (BioRad), and droplets generated on QX200 Automated Droplet Generator (BioRad) according to the manufacturer’s instructions. 5 ng of input FFPE tissue-derived DNA were analysed in each well. Each reaction was partitioned to a median of 16,000 droplets, including a tested sample (ran in duplicate), positive, negative controls and nontemplate control (nucleic acid-free water). Emulsified PCR reactions were run on a 96-well plate on a BioRad thermal cycler, incubating the plates at 95°C for 10 min, followed by 40 cycles at 95°C for 15 sec and 60°C for 60 sec, then by a 10 min incubation at 98°C. The temperature ramp increment was 2.5°C/sec for all steps. Plates were read on a BioRad QX200 droplet reader using QuantaSoft version 1.7.4.0917 software from BioRad. Number of droplets positive for mutant DNA, wild type DNA, both, or neither was assessed, and fractional abundance calculated for each well (or merged wells for tested sample).

### Statistical considerations

The sample size was limited by the number of patients who had undergone pulmonary metastasectomies and who had sufficient DNA for analysis. The majority of the analysis is descriptive. The frequency of alterations in each gene was calculated and the mutational profile of the colorectal primary tumour was compared to the profile of the metastases in order to determine the concordance between the samples.

The date of diagnosis of pulmonary metastatic disease was defined as the date PM were first detected by CT imaging. PFS was calculated from the date of diagnosis of pulmonary metastatic disease to the date of progression or death from any cause. OS was calculated from the date of diagnosis of pulmonary metastatic disease to the date of death from any cause. Patients without an event were censored at the time of last follow-up. Cox proportional hazards models were used to investigate whether the number of pulmonary metastasectomies (2+ vs. 1), pre-operative CEA (> 4 vs. ≤ 4), site of primary tumour (colon vs. rectum), size of largest metastasis (11 mm vs. ≤ 11 mm), number of metastases (> 1 vs. 1), site of PM (unilateral vs. bilateral), neoadjuvant chemotherapy (yes vs. no), adjuvant chemotherapy (yes vs. no), site of first metastasis (pulmonary only vs. other) and time to first metastasis were associated with PFS and OS.

## SUPPLEMENTARY MATERIALS FIGURES AND TABLES





## Abbreviations

ddPCR: digital droplet polymerase chain reaction
FFPE: formalin fixed paraffin embedded
IQR: interquartile range
NGS: next generation sequencing
PM: pulmonary metastasis
PFS: progression-free survival
RFA: radiofrequency ablation
OS: overall survival
VAF: variant allele frequency
