# Supplementary material for: Molecular profiling of colorectal pulmonary metastases and primary tumours: implications for targeted treatment
Source: Oncotarget. 2017 Apr 11;8(39):64999–5008. doi: 10.18632/oncotarget.17048 (PMC5630307; doi:10.18632/oncotarget.17048)
Supplement: Supplementary file 1 [file oncotarget-08-64999-s001.pdf]

Molecular profiling of colorectal pulmonary metastases and primary tumours: implications for targeted treatment

Supplementary Materials

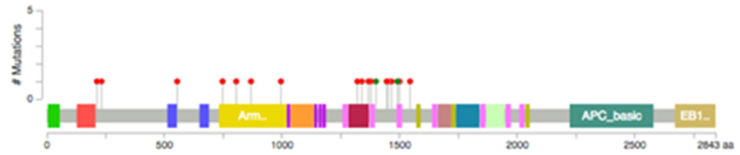

Sample ID	AA change		Type	COSMIC	End Pos	Ref
P002	*Arg805**		Nonsense		112173704	112173704
P011	*Arg554**	3D	Nonsense		112164586	112164586
P011	*Arg1399His*	3D	Missense		112175487	112175487
P011	*Val1320fs*		FS del		112175248	112175248
P025	*Gln1378**	3D	Nonsense		112175423	112175423
P032	*Gly1339fs*		FS del		112175307	112175307
P032	*Gln1367**	3D	Nonsense		112175390	112175390
P037	*Glu868**		Nonsense		112173893	112173893
P037	*Gly1499fs*	3D	FS del		112175787	112175787
P040	*Cys995**		Nonsense		112174276	112174276
P040	*Thr1445fs*	3D	FS del		112175625	112175625
P041	*Arg213**	3D	Nonsense		112116592	112116592
P041	*His1490Tyr*	3D	Missense		112175759	112175759
P050	*Arg232**	3D	Nonsense		112128191	112128191
P050	*Glu1544fs*	3D	FS del		112175920	112175920
P045	*Ser1465fs*	3D	FS del		112175675	112175675
P056	*Arg1450**	3D	Nonsense		112175639	112175639
P056	*Ser747**		Nonsense		112173531	112173531

Supplementary Figure 1: Mutations detected in *APC*.

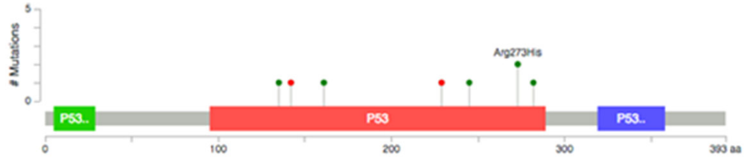

Sample ID	AA change		Type	COSMIC	End Pos	Ref
P007	*Ala161Asp*	3D	Missense		7578448	7578448
P011	*Arg282Trp*	3D	Missense		7577094	7577094
P032	*Pro142fs*	3D	FS del		7578504	7578504
P037	*Arg273His*	3D	Missense		7577120	7577120
P041	*Cys135Tyr*	3D	Missense		7578526	7578526
P050	*Arg273His*	3D	Missense		7577120	7577120
P052	*Gly245Ser*	3D	Missense		7577548	7577548
P056	*Cys229fs*	3D	FS del		7577593	7577593

Supplementary Figure 2: Mutations detected in *TP53*.

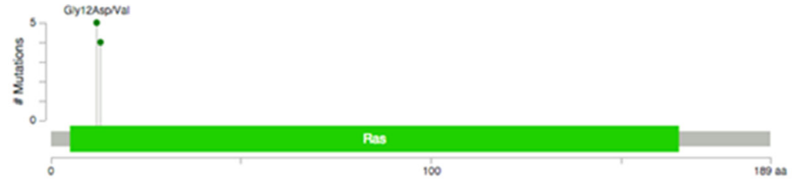

Sample ID	AA change		Type	COSMIC	End Pos	Ref
P002	Gly12Asp	3D	Missense		25398284	25398284
P011	Gly13Arg	3D	Missense		25398282	25398282
P025	Gly13Asp	3D	Missense		25398281	25398281
P032	Gly13Asp	3D	Missense		25398281	25398281
P037	Gly12Val	3D	Missense		25398284	25398284
P040	Gly12Val	3D	Missense		25398284	25398284
P050	Gly13Asp	3D	Missense		25398281	25398281
P052	Gly12Val	3D	Missense		25398284	25398284
P056	Gly12Val	3D	Missense		25398284	25398284

Supplementary Figure 3: Mutations detected in *KRAS*.

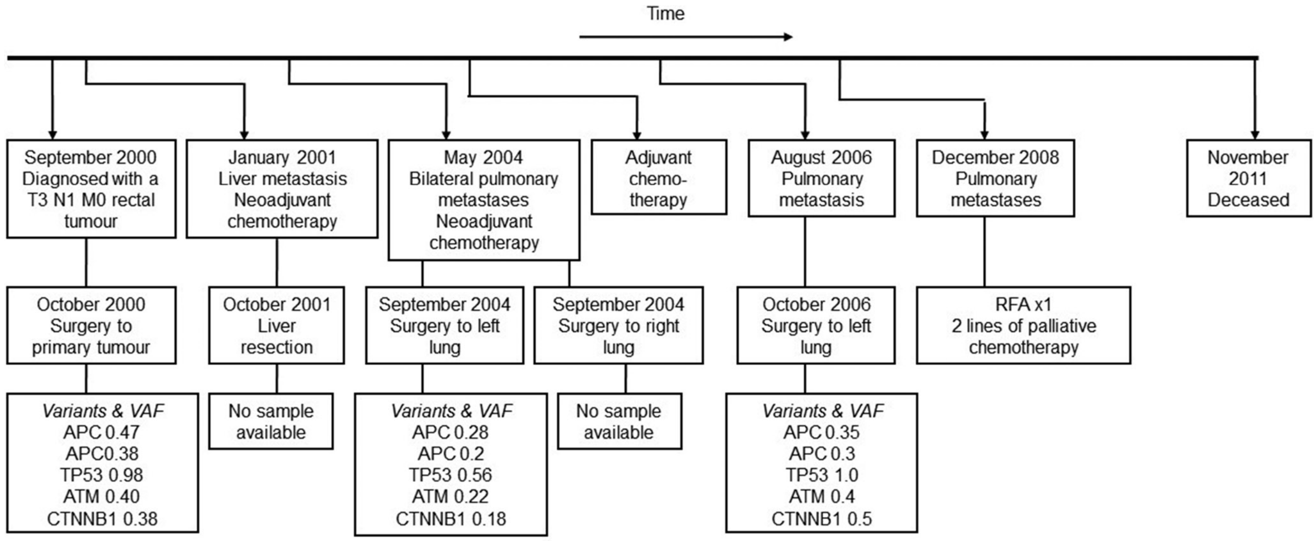

Supplementary Figure 4: Details of the treatment and molecular characteristics of patient 041. RFA = radiofrequency ablation, VAF = variant allele frequency.

Supplementary Table 1: Details of pulmonary metastasectomies (*n* = 121)

Characteristic	*N* (%)
Site of pulmonary metastases	
Unilateral	88 (73)
Bilateral	33 (27)
Neoadjuvant chemotherapy	
No	63 (52)
Yes	58 (48)
CAPOX/FOLFOX	29 (50)
CAPIRI/FOLFIRI	18 (31)
Raltitrexed and oxaliplatin	7 (12)
Other	4 (7)
Neoadjuvant targeted/anti-angiogenic therapy	
Bevacizumab	12 (10)
Cetuximab	1 (1)
Sunitinib	1 (1)
Median CEA prior to pulmonary metastasectomy (IQR)[1]	2 (2–4)
Number of histologically confirmed metastases (per metastasectomy)	
0[2]	6 (5)
1	67 (55)
2	26 (22)
3	9 (7)
4	5 (4)
≥ 5	8 (7)
Median diameter of largest resected pulmonary metastasis (IQR), mm	12 (9–15)
Post-operative chemotherapy	
No	67 (55)
Yes	53 (43)
CAPOX/FOLFOX	20 (38)
CAPIRI/FOLFIRI	14 (26)
Capecitabine/5-FU +/− mitomycin	13 (25)
Other	6 (11)
Unknown	1 (2)
Post-operative targeted/anti-angiogenic therapy	
Bevacizumab	12 (10)
Cetuximab	3 (3)

[1]Data only available for 68 of 121 metastasectomies. [2]These samples showed a chemotherapeutic response, with no viable tumour remaining.
CAPOX = capecitabine/oxaliplatin, FOLFOX = 5-fluorouracil/oxaliplatin, CAPIRI = capecitabine/irinotecan, FOLFIRI = 5-fluorouracil/irinotecan.

Supplementary Table 2: Variants detected in the first PM for each patient (*n* = 24).
See Supplementary_Table_2
